# A critical role of holistic processing in face gender perception

**DOI:** 10.3389/fnhum.2014.00477

**Published:** 2014-06-26

**Authors:** Takemasa Yokoyama, Yasuki Noguchi, Ryosuke Tachibana, Shigeru Mukaida, Shinichi Kita

**Affiliations:** ^1^Department of Psychology, Kobe UniversityKobe, Japan; ^2^Graduate School of Environmental Studies, Nagoya UniversityNagoya, Japan; ^3^Japan Society for the Promotion of ScienceTokyo, Japan; ^4^Faculty of Information Media, Hokkaido Information UniversityEbetsu, Japan

**Keywords:** amodal completion, holistic processing, electroencephalography, gender, stereoscopic depth manipulation

## Abstract

Whether face gender perception is processed by encoding holistic (whole) or featural (parts) information is a controversial issue. Although neuroimaging studies have identified brain regions related to face gender perception, the temporal dynamics of this process remain under debate. Here, we identified the mechanism and temporal dynamics of face gender perception. We used stereoscopic depth manipulation to create two conditions: the front and behind condition. In the front condition, facial patches were presented stereoscopically in front of the occluder and participants perceived them as disjoint parts (featural cues). In the behind condition, facial patches were presented stereoscopically behind the occluder and were amodally completed and unified in a coherent face (holistic cues). We performed three behavioral experiments and one electroencephalography experiment, and compared the results of the front and behind conditions. We found faster reaction times (RTs) in the behind condition compared with the front, and observed priming effects and aftereffects only in the behind condition. Moreover, the EEG experiment revealed that face gender perception is processed in the relatively late phase of visual recognition (200–285 ms). Our results indicate that holistic information is critical for face gender perception, and that this process occurs with a relatively late latency.

## Introduction

Face perception is one of the most important cognitive functions in humans. Faces can provide a vast amount of information, such as identity, emotion, and intention (Bruce and Young, 1986). A face also tells the gender of the person, and face gender perception is vital for survival, as this function contributes to mate selection.

Two competing hypotheses of face gender perception propose different mechanisms for this function. The first hypothesis proposes that the featural information of faces, such as the eyes and mouth, is critical for face gender perception; most studies on face gender perception support this hypothesis (Brown and Perrett, 1993; Yamaguchi et al., 1995; Gosselin and Schyns, 2001; Schyns et al., 2002; Dupuis-Roy et al., 2009). Typically, the facial stimuli used in those studies were partly hidden by a mask or incorporated some male and female facial parts while observers performed the gender judgment task. Those studies indicated that certain facial parts are sufficient for recognizing face gender and suggested that the eyes are a facial part that is essential for face gender judgment, perhaps because visual information of the eyes or gaze has a great influence on cognitive functioning, such as perception, conscious awareness, and attention (Conty et al., 2006; Senju and Johnson, 2009; Yokoyama et al., 2011, 2012, 2013, 2014). By contrast, the other hypothesis states that the holistic information of faces plays a key role in face gender perception. In this line of research, researchers used inversion and composite effects and concluded that holistic cues are essential for face gender perception (Baudouin and Humphreys, 2006; Zhao and Hayward, 2010). In this sense, there is no conclusive evidence regarding the mechanism underlying face gender perception.

Neuroimaging and neurophysiological studies have attempted to identify the neural mechanism involved in face gender perception. In neuroimaging studies, face gender perception was related to the occipital face area (OFA), lateral fusiform gyrus (LFG), and fusiform face area (FFA; Freeman et al., 2010; Wiese et al., 2012; Podrebarac et al., 2013). Initial perceptual representation regarding face gender is processed in the FFA and LFG (Freeman et al., 2010; Podrebarac et al., 2013). The OFA is involved in the initial processing stage of face perceptional network and spatial relation of faces (Wiese et al., 2012). Therefore, these face regions in the occipital and temporal lobes seem to play a key role in face gender perception. Conversely, the precise temporal dynamics of face gender perception remain unclear. Previous research indicated that relatively early event-related potential (ERP) components, such as P1 and N1, are associated with face gender perception (Su et al., 2013), whereas other research suggested that relatively later ERP components, such as P2, are related to this function (Mouchetant-Rostaing et al., 2000; Mouchetant-Rostaing and Giard, 2003; Ito and Urland, 2005). Resolving this debate and clarifying the temporal dynamics of neural activity would promote further understanding of the neural networks between the above-mentioned face-related regions (OFA, LFG, and FFA) identified by neuroimaging studies.

The aim of the current study was to identify the mechanism involved in face gender perception and to measure its corresponding temporal dynamics. Several previous studies that supported featural processing used facial stimuli that were partly hidden by a mask, whereas other studies used faces that combined male and female facial parts. In the former studies, because perceptual filling-in might have occurred, the participants should perceive the facial stimuli as complete faces. This is because, although an object is partly occluded by other objects, the occluded object is seen as a complete object (Nakayama et al., 1989). In the latter studies, even though facial stimuli incorporated some male and female parts, holistic cues remained; therefore, participants were able to use holistic information for face gender judgment. Thus, those studies cannot exclude the possibility that holistic cues are related to face gender. An alternative method to elucidate face gender perception is needed.

To resolve the methodological problem mentioned above, we took advantage of the phenomenon of amodal completion with stereoscopic depth manipulation (Nakayama et al., 1989; Fang and He, 2005; Hegdé et al., 2008; Chen et al., 2009). In this manipulation, identical fragments are presented either in front of or behind occluding visual noise. If the fragments are shown stereoscopically behind the occluder (behind condition), those fragments are amodally completed and unified into a coherent object by the participants. Conversely, if the same fragments are shown stereoscopically in front of the occluder (front condition), participants perceive them as disjoint parts and amodal completion does not occur. Participants can use only featural cues in the front condition, whereas they can use both featural and holistic cues in the behind condition. Therefore, if holistic cues are essential for face gender perception, performance will be higher in the behind condition than in the front. In addition, because it is controversial whether the early or late ERP component plays a more important role in face gender perception, we used amodal completion with EEG to resolve this debate.

The current study examined which type of processing (featural or holistic) is involved in face gender perception. We conducted three behavioral experiments and one EEG experiment. First, we simply compared the reaction times (RTs) of gender judgment between the front and behind conditions (Experiment 1). To consolidate the differences between the front and behind conditions, we compared them further using paradigms of priming effects (Experiment 2A) and negative aftereffects (Experiment 3). Finally, we performed an EEG experiment and compared repetition suppression between the two conditions (Experiment 2B).

## Experiment 1

We first performed a simple experiment to test which type of processing (holistic or featural) was critical for face gender perception using stereoscopic depth manipulation (manipulation of the depth position of a target face presented in front of or behind the occluder). Participants perceived disjointed patches when the same patches were presented stereoscopically in front of the occluder (front condition). By contrast, participants perceived a coherent face when the face patches were presented stereoscopically behind the occluder (behind condition). Thus, the front condition had only featural cues of faces, whereas the behind condition had both featural and holistic cues. The hypothesis behind Experiment 1 was that, if the holistic information of a face is critical for the judgment of face gender, RTs in the behind condition should be faster than RTs in the front condition. Otherwise, we should observe no differences between the two conditions because both conditions have featural information.

### Method

#### Participants

Six participants (4 males, 2 females; age range, 21–26 years) were recruited from Kobe University. Each participant gave informed consent after the nature of the study was explained. All participants had normal or corrected-to-normal visual acuity, and all were naïve to the purposes of this experiment. Approval for the experiment was obtained from the ethics committee of the Department of Psychology, Kobe University, Kobe, Japan.

#### Apparatus

Visual stimuli were displayed on a Sony MultiScan 17sf II 14.1-inch CRT display with a resolution of 1024 × 768 pixels. Displays and data collection were controlled using the Psychophysics Toolbox of MATLAB (Brainard, 1997; Pelli, 1997) on a Dell Optiplex 360 computer under Microsoft Windows XP (refresh rate was 60 Hz). The viewing distance was 31 cm.

#### Stimuli and procedure

**Figure 1:**
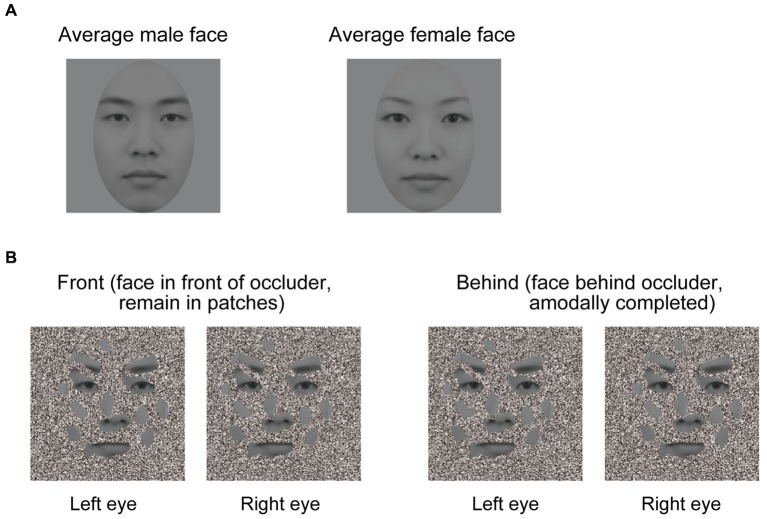
**Visual stimuli used in the experiments. (A)** Averaged faces. The left panel denotes an averaged male face, and the right panel denotes an averaged female face. Sixty male and female faces were used to create averaged male and female faces. **(B)** Schematic illustration of the experimental conditions (the front and behind conditions). A face is perceived as a complete face when the occluder is in the near depth plane (left panel), but not in the far depth plane (right panel), even though the stimuli presented in the front and behind conditions were identical. In the right panel, face patches appear like a collection of disjointed face patches hovering in depth. A good example of this is Figure 1B in Hegdé et al. (2008).

To create dichoptic viewing, visual stimuli were presented at two different locations on the screen. Two white frames (12.8 × 12.8° of visual angle) were displayed side by side on the screen, with one frame presented to each eye. Stimuli at those two locations were fused by a mirror stereoscope. We used 60 male and female faces to create morphed average faces for each gender (Figure 1A). These average male and female faces were presented in gray scale. The facial images comprised a neutral facial expression because emotional expressions appear to interact with face gender perception (Hess et al., 2009; Valdés-Conroy et al., 2014). The occluder was an orthonormal planar surface containing random and irregular holes (Figure 1B), and about 43% of the facial area was visible through its holes. The visible facial area included the eyes, nose, mouth, and eyebrows. Face patches were always at 0 disparity, and the occluder was at either +0.267° or −0.267° of arc disparity.

Experiment 1 had a one-factorial repeated-measures design with two levels: the front and behind conditions. Four experimental blocks of 30 trials were conducted, with 60 trials for each condition. Male and female pictures were intermixed randomly in a block. Each condition was not intermixed in a block, and a Latin square was used to prevent practice effects. The experimental trials were preceded by 20 practice trials, and the order of blocks separated by each condition was randomized across participants. Feedback was not provided in either the practice or experimental trials. Each trial cycled through the fixation display and was followed by a facial stimulus, and the facial stimulus was presented until the participant responded. Participants were required to press the right (female) or left (male) key as quickly and accurately as possible.

To evaluate a contribution of the featural and holistic cues in face gender perception, we used pairwise *t-*tests to compare the RTs of performance between the front and behind conditions. If the holistic cues played a critical role in face gender perception, we should observe faster RTs in the behind compared with the front condition.

### Results

**Figure 2:**
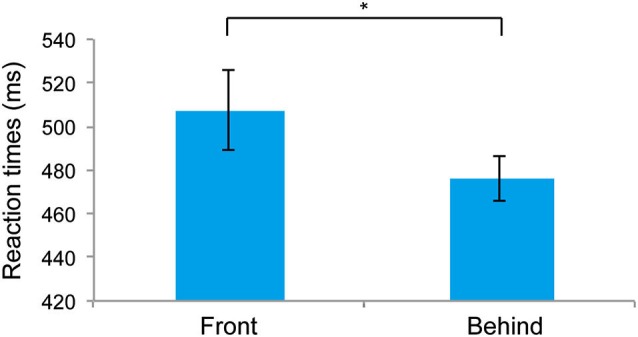
**Mean reaction times (RTs) recorded in Experiment 1**. The error bars represent the standard error of the mean. * *P* < 0.05.

Figure 2 shows the RTs for the front and behind conditions. RTs were significantly faster for the behind than for the front conditions (*t*_5_ = 3.429, *P* = 0.018). The mean (± SE) error rates of the front and behind conditions were 5.27 ± 1.09% and 3.33 ± 0.96%, respectively, and these differences were not significant (*t*_5_ = 1.659, *P* = 0.157). The observation of faster RTs in the behind condition compared with the front condition suggests that the holistic information of faces is critical for the judgment of face gender. To test the possible influence of the subjects’ gender on face perception (Cellerino et al., 2004; Sun et al., 2010), we also analyzed the RT data by combining the factor of stereoscopic depth (front/behind) with that of the subjects’ gender (male/female). Two-way ANOVA showed a nonsignificant main effect of the subjects’ gender (*F*_(1, 4)_ = 0.599, *P* = 0.482), indicating that the subjects’ gender did not affect the RTs in the present study.

The perceived depth position of a target face differed between the front and behind conditions in Experiment 1. Therefore, one might argue that the results of Experiment 1 could have been caused by the perceived depth position. To exclude this possibility, we conducted a priming experiment, in which two stimuli (a prime and a target) were presented sequentially, and participants responded to the target. In this type of experiment, when stereoscopic depth manipulation is used only for the prime, the response to the target is not influenced by the perceived depth position. Thus, the priming paradigm can exclude the possible influence of the perceived depth position of a target face and should help consolidate the results obtained in Experiment 1.

In addition to the behavioral experiment, we performed an EEG experiment to examine the temporal dynamics of face gender perception using a similar priming paradigm. It is well known that when observers see a visual stimulus, the neural activity related to the stimulus is decreased if the stimulus had been presented previously (Desimone, 1996; Oldenburg et al., 2012). This neural characteristic is the so-called repetition suppression. To take advantage of this phenomenon, we examined the temporal dynamics of face gender perception.

## Experiment 2A

We conducted the priming experiment in Experiment 2 to exclude the possible influence of perceived depth position of a target face, and to consolidate the results obtained in Experiment 1. We prepared two factors: congruency (congruent and incongruent conditions) and a prime (no occluder, 2D, front condition, and behind condition). If the priming effect is observed in both the front and behind conditions, then the results obtained in Experiment 1 can be attributed to the perceived depth position of a target face. Conversely, if the priming effect is observed in the behind condition exclusively, the possibility of perceived depth position can be excluded, and the results strengthen those obtained in Experiment 1.

### Method

Experiment 2A was identical to Experiment 1, with the exception of the following details.

#### Participants, stimuli, and procedure

Twelve participants (9 males, 3 females; age range, 20–31 years) participated in Experiment 2A. Experiment 2A had a 2 × 4 repeated-measures design. The first factor was congruency (congruent and incongruent), and the second factor was a prime (front, 2D, no occluder, and behind). The prime and target were of the same gender in the congruent condition but were of different genders in the incongruent condition. Unlike what was observed in the front and behind conditions, in the 2D condition, the prime occluder did not exhibit disparity, indicating that the participants did not perceive a 3D image in this condition. In the no-occluder condition, average male or female faces without occluder were presented. The target image was an intact average male or female face. Four experimental blocks of 72 trials were conducted, with 36 trials for each condition. The congruency factor was intermixed randomly in a block, whereas the prime factor was separated into a block and randomized across participants. The experimental trials were preceded by 20 practice trials, and the order of blocks separated by the condition was randomized across participants. Each trial cycled through the fixation display and was followed by the presentation of a prime display for 500 ms. Subsequently, a blank display was presented for 100 ms, and the target facial stimulus was presented until the participant responded. Participants were required to press the right (female) or left (male) key as quickly and accurately as possible.

To evaluate the priming effects, we used pairwise *t-*tests to compare the RTs of performance in the congruent and incongruent conditions. We also performed one-factorial repeated measures ANOVA to examine the priming effects of the four prime types. If the holistic cues played a critical role in face gender perception, we should observe priming effects in the behind condition but not in the front condition.

### Results

**Figure 3:**
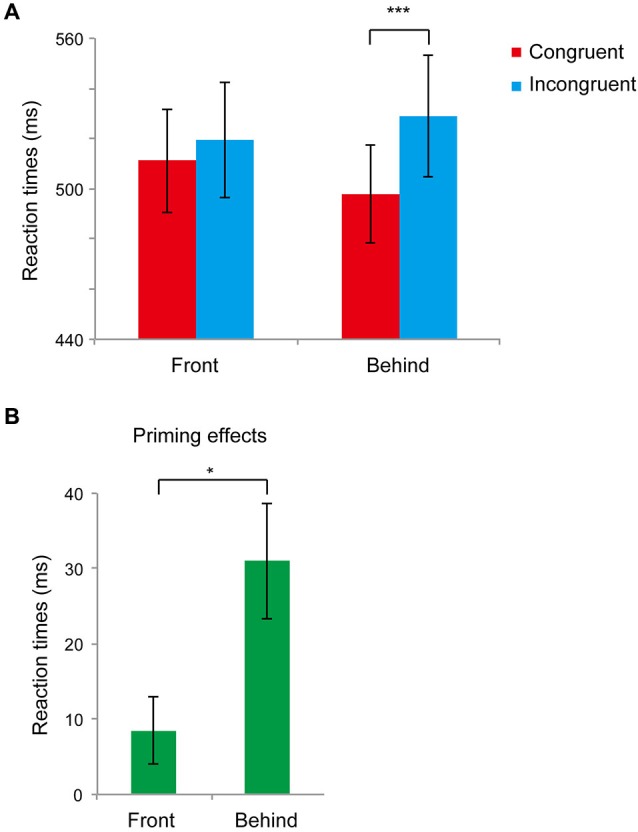
**Results of Experiment 2. (A)** Mean reaction times (RTs) recorded in Experiment 2 in the front and behind conditions. The red bar represents the congruent condition (the face gender of the prime and target was the same); the blue bar represents the incongruent condition (the face gender of the prime and target was different). Bars represent standard errors (SEs) across participants. **(B)** Congruency effects (incongruent–congruent) of RTs in the front and behind conditions. Bars represent SE between participants. * *P* < 0.05, *** *P* < 0.005.

Figure 3A shows the main results (RTs for the front and behind conditions) of Experiment 2. First, we performed a paired *t-*test analysis in the front and behind conditions to analyze priming effects (difference between the RTs of the congruent and incongruent conditions). We found significant differences between the congruent and incongruent conditions in the behind condition (*t*_11_ = 4.03, *P* = 0.002) but not in the front condition (*t*_11_ = 1.90, *P* = 0.083). In the front condition, the error rates were 0.027 ± 0.01% and 0.032 ± 0.01% in the congruent and incongruent conditions, respectively, and the difference between conditions was not significant (*t*_5_ = 0.431, *P* = 0.674). In the behind condition, the error rate was larger for the incongruent condition (0.05 ± 0.01%) compared with that of the congruent condition (0.02 ± 0.01%) (*t*_5_ = 3.369, *P* = 0. 006). We next performed a paired *t-*test to compare the priming effects in these conditions. The priming effects differed significantly between the front and behind conditions (*t*_11_ = 3.09, *P* = 0.010; Figure 3B).

To evaluate whether there were differences between male and female subjects, we performed a 2 (men and women: sex) × 2 (congruent and incongruent: congruency) mixed ANOVA in each front and behind condition, and we found no sex difference in either condition (front: *F*_(1, 10)_ = 0.709, *P* = 0.419; behind: *F*_(1, 10)_ = 0.400, *P* = 0.541). A subsequent one-factorial repeated-measures ANOVA of the RTs revealed a significant main effect of prime type (*F*_(3, 33)_ = 5.065, *P* = 0.005). A *post hoc* comparison using the Ryan method confirmed the presence of a significant difference between the no-occluder and 2D conditions (*t*_33_ = 2.957, *P* = 0.005), the no-occluder and front conditions (*t*_33_ = 2.774, *P* = 0.009), the behind and 2D conditions (*t*_33_ = 2.723, *P* = 0.010), and the behind and front conditions (*t*_33_ = 2.540, *P* = 0.015). We observed priming effects for the behind condition but not for the front condition. These results consolidated the results of Experiment 1 and suggest that holistic information plays a key role in face gender judgment, regardless of the use of stereoscopic vision.

## Experiment 2B

### Method

Experiment 2B was identical to Experiment 2A, with the exception of the following details.

#### Participants, stimuli, and procedure

Twelve participants (9 males, 3 females; age range, 20–31 years) participated in Experiment 2B. Eight experimental blocks of 72 trials were conducted, with 72 trials for each condition. Each trial cycled through the fixation display, followed by the presentation of a prime display for 500 ms, after which a blank display was presented for 300 ms, and the target facial stimulus was presented for 500 ms. After the trial, participants pressed one key when the target face was male and another key when the target face was female.

#### EEG measurement and data analysis

In Experiment 2B, we acquired electroencephalograms from 19 scalp positions from the International 10–20 system: FP1, FP2, F3, Fz, F4, F7, F8, C3, Cz, C4, T3, T4, T5, T6, P3, Pz, P4, O1, and O2 (EEG1200, Nihon Kohden, Tokyo, Japan). EEG was recorded continuously at a rate of 500 Hz, and was referenced with an average potential measured from the right and left earlobes. Interelectrode impedance was kept below 5 kΩ. Neural activities in response to the facial images were examined by measuring visual-evoked potentials (VEPs) that were time-locked to the onset of those images. We epoched and averaged EEG waveforms that ranged from −900 to 500 ms (0 ms is the onset of the target image, not prime image).

Epochs with noisy EEG waveforms were excluded from the analyses by the following procedures. First, we applied a high-pass filter of 0.1 Hz to raw (pre-average) EEG waveforms, correcting for slow drifts in those data. Using those filtered waveforms, we searched for noisy epochs in which a variation in EEG data (a difference between maximum and minimum values within an epoch) exceeded 100 μV. Epochs with large variations (caused by body motion, eye blinks, and eye movements, etc.) were thus identified as noisy epochs. We then performed the across-trial (across-epoch) averaging of EEG data with the noisy epochs (identified in a previous step) excluded from the averaging. To avoid possible bias on the waveforms caused by the high-pass filtering (Acunzo et al., 2012; Rousselet, 2012), this across-trial averaging were conducted on the pre-filtered data (the raw EEG waveforms before the high-pass filter of 0.1 Hz had been applied). Finally, we applied a low-pass filter of 30.0 Hz to the post-average EEG waveforms (VEPs). The numbers of averaged epochs out of 72 possible epochs were 63.6 (no occluder with congruent condition), 63.5 (no occluder with incongruent condition), 62.9 (2D with congruent condition), 61.8 (2D with incongruent condition), 59.4 (front with congruent condition), 57.8 (front with incongruent condition), 53.6 (behind with congruent condition), and 53.3 (behind with incongruent condition).

**Figure 4:**
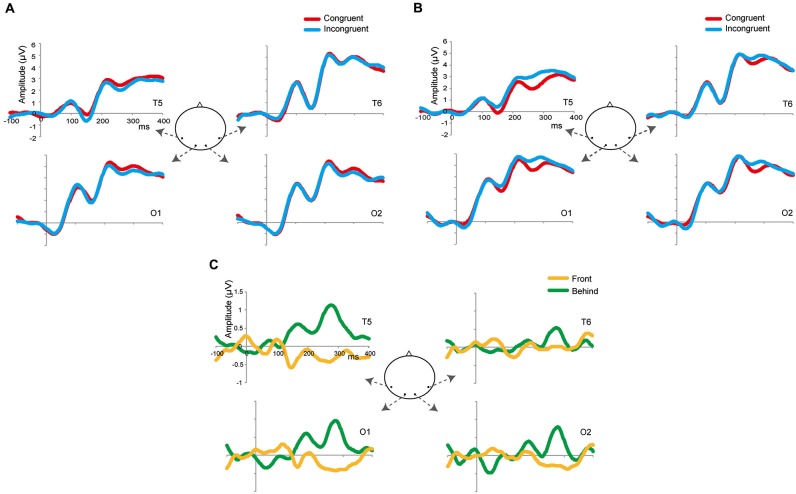
**Grand-average ERPs in the front (A) and behind (B) conditions at four selected occipitotemporal electrodes (T5, T6, O1, and O2)**. The zeros in the horizontal axis denote the onset of the presentation of target images (and not of prime images). The red waveforms denote the congruent condition; the blue waveforms denote the incongruent condition. **(C)** Grand-average subtracting ERPs (incongruent–congruent). The yellow waveforms denote the front condition; the green waveforms denote the behind condition.

Although we made the factors and conditions identical to those used in Experiment 2A, our main interest was to identify differences between the front and behind conditions; thus, we focused on the analysis of these two conditions. We analyzed latencies and mean amplitude of the P1, N1, and P2 components. To identify the time windows of these three components, we used the method of Keyes et al. (2010) (P1: 90–145 ms, N1: 145–200 ms, P2: 200–285 ms). To identify the electrodes related to face gender perception, we used the method of Mouchetant-Rostaing and Giard (2003). We also used repetition suppression as an index of face gender perception. Thus, we used the subtracted ERP (incongruent—congruent; Figure 4C) and channels to perform repeated-measures ANOVA to analyze the mean amplitude in each component. In the analysis of latencies of these components, we used congruency (congruent and incongruent) and channels in the repeated-measures ANOVA.

Based on the results of Experiment 2, we expected the ERP amplitude and/or latencies in the behind condition to be smaller and/or faster than those observed in the front condition (repetition suppression). Therefore, if the early ERP component is related to face gender perception, repetition suppression should be larger for the behind condition than for the front condition in the P1 and/or N1 component; otherwise, we should observe these differences in the P2 component.

### Result: conventional analysis of ERP components

#### Mean amplitude

**Figure 5:**
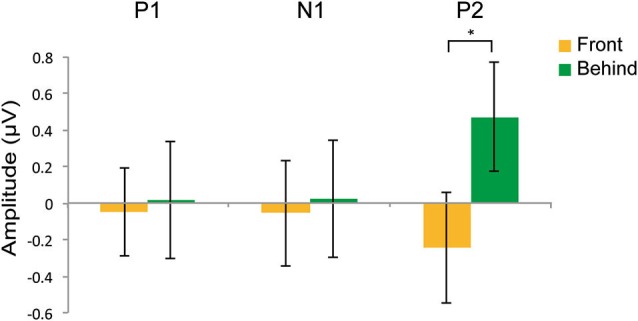
**Effects on repetition suppression in the EEG study**. The epoch of P1was 90–145 ms; the epoch of N1 was 145–200 ms; the epoch of P2 was 200–285 ms. The error bars represent the standard error of mean. * *P* < 0.05.

Figures 4A,B shows the grand-averaged ERPs at the occipitotemporal channels (O1, O2, T5, and T6) in the front and behind conditions, respectively. We conducted a 2 (front/behind: depth) × 4 (channel) repeated-measures ANOVA of the subtracted ERP amplitude in each P1, N2, and P2 component. In the P1 component, there were no significant main effects of depth (*F*_(1, 95)_ = 0.046, *P* = 0.834) or channel (*F*_(3, 95)_ = 0.326, *P* = 0.806), and no interaction (*F*_(3, 95)_ = 0.391, *P* = 0.760). In the N1 component, there were no significant main effects of depth (*F*_(1, 95)_ = 0.065, *P* = 0.803) or channel (*F*_(3, 95)_ = 0.190, *P* = 0.902), and no interaction (*F*_(3, 95)_ = 2.718, *P* = 0.060). In the P2 component, there was a significant main effect of depth (*F*_(1, 95)_ = 5.513, *P* = 0.038) but not of channel (*F*_(3, 95)_ = 0.375, *P* = 0.771), and no interaction (*F*_(3, 95)_ = 2.138, *P* = 0.114). Repetition suppression was larger in the behind condition than in the front condition in the P2 component (Figure 5). These EEG results also support the contention that holistic information about faces is critical to face gender judgment and that this process occurs in the later ERP component.

#### Peak latencies

##### Front condition

We applied 2 (congruent/incongruent: congruency) × 4 (channel) repeated-measures ANOVA to the front condition in each P1, N1, and P2 component. In the P1 component, there were no significant main effects of congruency (*F*_(1, 95)_ = 0.067, *P* = 0.800) or channel (*F*_(3, 95)_ = 2.429, *P* = 0.082), and no interaction (*F*_(3, 95)_ = 0.694, *P* = 0.562). In the N1 component, there were no significant main effects of congruency (*F*_(1, 95)_ = 2.235, *P* = 0.163) or channel (*F*_(3, 95)_ = 0.770, *P* = 0.518), and no interaction (*F*_(3, 95)_ = 1.179, *P* = 0.332). In the P2 component, there were no significant main effects of congruency (*F*_(1, 95)_ = 0.007, *P* = 0.933) or channel (*F*_(3, 95)_ = 0.035, *P* = 0.991), and no interaction (*F*_(3, 95)_ = 0.302, *P* = 0.823). Consequently, there were no differences in latencies between the congruent and incongruent conditions in these components in the front condition.

##### Behind condition

We applied 2 (congruent/incongruent) × 4 (channel) repeated-measures ANOVA to the behind condition in each P1, N1, and P2 component. In the P1 component, there were no significant main effects of congruency (*F*_(1, 95)_ = 0.570, *P* = 0.466) or channel (*F*_(3, 95)_ = 0.716, *P* = 0.549), and no interaction (*F*_(3, 95)_ = 0.833, *P* = 0.485). In the N1 component, there were no significant main effects of congruency (*F*_(1, 95)_ = 0.570, *P* = 0.466) or channel (*F*_(3, 95)_ = 0.716, *P* = 0.549), and no interaction (*F*_(3, 95)_ = 0.833, *P* = 0.485). In the P2 component, there were no significant main effects of congruency (*F*_(1, 95)_ = 0.200, *P* = 0.663) or channel (*F*_(3, 95)_ = 1.294, *P* = 0.292), and no interaction (*F*_(3, 95)_ = 0.101, *P* = 0.958). Consequently, there were no differences in latencies between the congruent and incongruent conditions in these components in the behind condition.

## Experiment 3

Neural systems have two properties: excitation and inhibition. The priming effect observed in Experiment 2A reflects the excitatory property of the neural systems. We next investigated whether holistic information influences the inhibitory property of neural systems during face gender perception. To address this question, we used adaptation. Adaptation is a typical property of almost all neural systems that isolates and temporarily reduces the contribution of specific neural populations (Webster et al., 2004; Fang and He, 2005). Thus, we reasoned that an adaptation experiment would permit the measurement of the inhibitory property of the neural systems involved in face gender processing.

The magnitudes of the adaptation were evaluated by measuring changes in the point of subjective equality (PSE; 50% threshold) of curves. Adaptation to female faces induces observers to judge the following faces as more likely to be male faces and vice versa (Kloth et al., 2010). Thus, if aftereffects occur, the female adaptor condition should increase “male” responses at several levels of morph range, resulting in a leftward shift of the sigmoid function and a lower PSE. By contrast, the male adaptor condition should induce a rightward shift of the function and a greater PSE.

### Method

Experiment 3 was identical to Experiment 1, with the exception of the following details.

#### Participants, stimuli, and procedure

Six participants (4 males, 2 females; age range, 21–31 years) participated in Experiment 3. Test stimuli were morphed between the average male and female faces. The morph range had seven levels: 100% male (0% female), 83% male (17% female), 67% male (33% female), androgynous, 33% male (67% female), 17% male (83% female), and 0% male (100% female). The adapting stimuli were average male or female faces with occluders and disparity.

We set up two separate sessions: adaptation and baseline sessions. The adaptation session was devoted to trials in which the adapting and test stimuli were presented, and the baseline session was devoted to trials in which only the test stimulus was presented. The baseline session was performed first, and the adaptation session was performed at least 1 week after the baseline session was performed.

In the adaptation session, six blocks of 40 trials were conducted. There were two conditions (front and behind conditions), and each condition comprised 120 trials. Trials with 100% male and female test stimuli were presented 10 times, and those with other test stimuli (83% male, 67% male, androgynous, 33% male, and 17% male) were presented 20 times. The experimental trials were preceded by 10 practice trials for each condition. Each trial cycled through the fixation display, followed by the presentation of an adapting stimuli for 5 s. Subsequently, test stimuli were presented for 250 ms. After the trial, participants pressed one key when the target face was male and another key when the target face was female.

In the baseline session, three blocks of 40 trials were conducted. The number of presenting test stimuli was the same as in the adaptation session. Ten practice trials were conducted before the experimental trials. Each trial cycled through the fixation display, followed by the presentation of a gray screen with white frames for 5 s. Subsequently, the test stimuli were presented for 250 ms. The task was the same as in the adaptation session.

For each condition and each participant, the changes in the percentage of “male” responses as a function of the seven levels of morph ranges were fitted using the sigmoid psychophysical function (Noguchi et al., 2011; Suzuki et al., 2013) as follows:

$$F(\chi )=Min+(Max-Min)/[1+e^{-a(\chi -b)}],$$

where χ is the morph range and *a* and *b* are free parameters estimated using the Nelder–Mead method (Nelder and Mead, 1965). Min and Max denote the minimum and maximum percentage of “male” responses throughout the seven levels of morph ranges, respectively.

To evaluate the aftereffects, we first conducted a pairwise *t-*test analysis between 0 and PSEs (between female and male adaptor conditions) in each of the front and behind conditions. We also performed pairwise *t*-test analysis on the Δ thresholds (male adaptor—female adaptor) between the front and behind conditions to compare these two directly. If the holistic cues played a critical role in face gender perception, we should observe aftereffects only in the behind condition.

### Result

**Figure 6:**
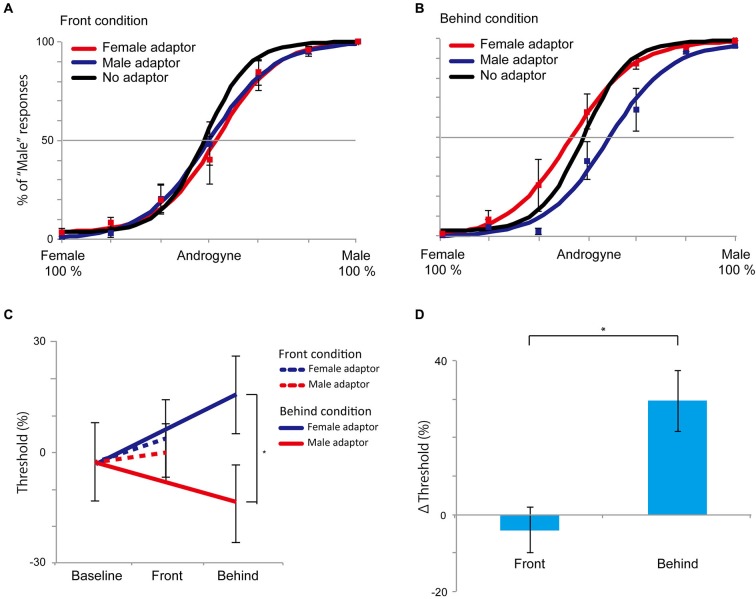
**Results of Experiment 3. (A and B)** The front and behind conditions in Experiment 3. Compared with the control (no adaptor) condition, there were no perceptual biases in the front condition. In the behind condition, the female adaptor, however, induced an increase in the percentage of “male” at several levels of morph ranges, while the percentage of “male” was decreased in the male adaptor condition. **(C)** PSEs in Experiment 3. The dotted line denotes the front condition; the straight line denotes the behind condition. **(D)** Magnitudes of the aftereffects as measured by differences in PSEs between female and male adaptors in the front and behind conditions. All error bars represent standard errors (SEs) across participants. * *P* < 0.05.

The results of Experiment 3 are shown in Figure 6. In the front condition, both curves of the female and male adaptor trials were similar to the baseline condition. Conversely, in the behind condition, the female adaptor trials generally had a higher percentage of “male” responses across the several levels of morph range, whereas the male adaptor trials had a lower percentage of “male” response percentages compared with baseline trials. The mean (± SE) magnitude of adaptation in the behind condition, as quantified based on the differences in the PSEs between the female and male adaptor conditions, was 29.5% (± 7.9%), which was significantly larger than 0 (*t*_5_ = 3.74, *P* = 0.003, one-group *t*-test; Figure 6C). By contrast, there was no significant difference in the front condition (−4.0% (± 5.9%), *t*_5_ = 0.677, *P* = 0.513, one-group *t*-test). In addition, a direct comparison between the front and behind conditions revealed a significant difference in the Δ thresholds (*t*_5_ = 2.887, *P* = 0.034, paired *t*-test; Figure 6D), indicating that the bias in the perceived face adaptor was substantially diminished in the front condition. To evaluate whether there were sex differences, we conducted 2 (men and women: sex) × 2 (front and behind: depth) mixed ANOVA, and we found no sex differences (*F*_(1, 4)_ = 0.105, *P* = 0.762). The results of Experiment 3 also supported the hypotheses that the holistic information of faces plays a key role in face gender judgment and that holistic information influences the inhibitory property of neural systems during face gender perception.

## Discussion

In this study, we investigated which type of information of faces (holistic or featural) plays a key role in face gender perception. The findings from our four experiments consistently support the hypothesis of holistic processing. In Experiment 1, participants recognized face gender more quickly when a face had holistic cues than when a face had featural cues. In Experiment 2A (priming experiment), we observed priming effects only in the behind condition. In Experiment 3 (adaptation experiment), aftereffects were observed only when holistic information was available. In addition to behavioral experiments, we used the priming paradigm and measured the temporal dynamics of face gender perception in Experiment 2B. The repetition suppression was larger for the behind than for the front condition in mean amplitude of the P2 components. Taken together, these results indicate that holistic processing plays a key role in face gender perception and that this process seems to occur at a relatively late component.

### A mechanism of face gender perception

The current study demonstrated clearly and consistently that holistic representation is necessary for face gender perception. In previous studies that supported featural representation, facial stimuli were partly hidden by a mask. Because this manipulation could have caused 2D amodal completion, the observers in those studies should have perceived those facial parts as a complete face and were thus able to judge face gender correctly. However, if face gender perception occurs only via the encoding of featural cues, the priming effects and negative aftereffects should be observed in the front condition; however, we did not observe such effects in the front condition. Consequently, having only featural cues might be insufficient for face gender perception, whereas representation of holistic information is essential for face gender perception.

Even though the RTs were faster for the behind condition than for the front condition in Experiment 1, there were no significant differences between errors in the front and behind conditions (front: 5.27 ± 1.09%); behind, 3.33 ± 0.96%; pairwise *t*-test: *t*_5_ = 1.659, *P* = 0.157). Thus, participants were fairly accurate at recognizing face gender despite the exclusive presentation of featural cues. How did they judge gender by seeing just facial parts? When the featural information of faces is encoded, the representation of featural information accesses holistic representation in memory (Rhodes et al., 1993). Therefore, this access to memory probably enables the judgment of gender despite the exclusive presentation of featural cues.

### Temporal dynamics of face gender perception

Our EEG data indicate that face gender is processed at a relatively late component, a finding that is consistent with previous studies. Previous neurophysiological studies did not report an effect of gender processing on the N170 component, whereas such effects were found at a relatively late component, similar to our results (Mouchetant-Rostaing et al., 2000; Mouchetant-Rostaing and Giard, 2003; Ito and Urland, 2005). Face-specific N1 (N170) is related to the detection of faces (Bentin et al., 1996; Itier and Taylor, 2004), whereas later components, such as P2 and P3, are associated with social categorization and are sensitive to gender (Mouchetant-Rostaing et al., 2000; Ito and Urland, 2005). Given this evidence, a neural processing for face gender perception should occur at a later stage compared with that for face detection.

Our EEG data are also consistent with the theoretical model proposed by Bruce and Young (1986). This model assumed that two distinct modules are associated with face processing: a structural encoding module and a directed visual encoding module. The structural encoding module is related to processing facial features, and the directed visual encoding module is associated with social category information such as race and gender. This model also suggests that facial structural encoding precedes the processing of social category information. In our EEG data, the effect of gender processing occurred in the later components. Our results thus support the model of Bruce and Young from the viewpoint of temporal dynamics.

## Conclusion

In conclusion, we have described the mechanism involved in face gender perception and its temporal dynamics. Holistic processing is essential for face gender perception. This process occurs in a later ERP component such as P2 (200–285 ms after stimulus presentation). We believe that the current study provides important clues for further understanding of the mechanism underlying face gender processing.

## Funding

This study was supported by a grant to the first author (Takemasa Yokoyama) from the Grant-in-Aid for JSPS Fellows [11J02271], the second author (Yasuki Noguchi) from the Grant-in-Aid for Young Scientists (A) [22680022], the forth author (Shigeru Mukaida) from the Grant-in-Aid for Scientific Research (C) [23500263], and the last author (Shinichi Kita) from the Strategic Information and Communications R and D Promotion Programme (SCOPE) [101707012].

## Conflict of interest statement

The authors declare that the research was conducted in the absence of any commercial or financial relationships that could be construed as a potential conflict of interest.
